# R_0_ Estimation for the African Swine Fever Epidemics in Wild Boar of Czech Republic and Belgium

**DOI:** 10.3390/vetsci7010002

**Published:** 2019-12-27

**Authors:** Andrea Marcon, Annick Linden, Petr Satran, Vincenzo Gervasi, Alain Licoppe, Vittorio Guberti

**Affiliations:** 1 ISPRA Istituto Superiore per la Ricerca e la Protezione Ambientale, 40064 Ozzano E. (BO), Italy; vincent.gervasi@gmail.com (V.G.); vittorio.guberti@isprambiente.it (V.G.); 2 FARAH Research Center, Faculty of Veterinary Medicine, University of Liège, 4000 Liège, Belgium; a.linden@uliege.be; 3 State Veterinary Administration of the Czech Republic, 100/7 Slezská, Prague, Czech Republic; p.satran@svscr.cz; 4 Department of Environmental and Agricultural Studies, Public Service of Wallonia, 5030 Gembloux, Belgium; alain.licoppe@spw.wallonie.be

**Keywords:** African swine fever, R_0_, doubling time, eradication strategies, wild boar, Europe

## Abstract

African swine fever (ASF) is a contagious haemorrhagic fever that affects both domesticated and wild pigs. Since ASF reached Europe wild boar populations have been a reservoir for the virus. Collecting reliable data on infected individuals in wild populations is challenging, and this makes it difficult to deploy an effective eradication strategy. However, for diseases with high lethality rate, infected carcasses can be used as a proxy for the number of infected individuals at a certain time. Then R_0_ parameter can be used to estimate the time distribution of the number of newly infected individuals for the outbreak. We estimated R_0_ for two ASF outbreaks in wild boar, in Czech Republic and Belgium, using the exponential growth method. This allowed us to estimate both R_0_ and the doubling time (T_d_) for those infections. The results are R_0_ = 1.95, T_d_ = 4.39 for Czech Republic and R_0_ = 1.65, T_d_ = 6.43 for Belgium. We suggest that, if estimated as early as possible, R_0_ and T_d_ can provide an expected course for the infection against which to compare the actual data collected in the field. This would help to assess if passive surveillance is properly implemented and hence to verify the efficacy of the applied control measures.

## 1. Introduction

African swine fever (ASF) is a contagious haemorrhagic fever that affects both domesticated and wild pigs. ASF was introduced in Georgia in 2007 [1] and from there, in about a decade, it spread to other Caucasian and Eastern European countries [2] and to a large part of Eurasia including Southeast Asia [3]. Since no vaccine exists, the only strategy to limit the spread of the disease is to isolate the infected area. Stamping out of infected domestic pigs is planned and, in some cases, a preventive depopulation has been implemented in the whole infected area. Moreover, movements of domestic pigs and their products are forbidden. Therefore, not only the disease itself but also the control measures cause large economic losses to the pig industry of any infected country [4]. The control/eradication of ASF becomes even more difficult when wild boar is involved, as occurred in several European Union countries. In Europe the virus shows two main epidemiological reservoirs: wild boar (i.e., Baltic countries) and backyard pigs (i.e., Danube Delta), with several mixed situations [5]. The presence of the virus in wild boar populations represents a risk for the infection in domestic pigs, therefore eradicating the virus from the wild boar population became a priority for the pig sector of any infected country. When the ASF virus (ASFV) is introduced in a wild boar population, an epidemic wave is observed; while the wave spreads in space and time it leaves behind an endemic situation that can last for years. As a matter of fact, at the time of writing only Czech Republic has been able to eradicate ASF in the wild, whereas the other EU countries are still facing the presence of the virus in their wild boar populations (Belgium, Bulgaria, Estonia, Hungary, Latvia, Lithuania, Poland, Romania, and Slovakia). Since the appearance of the infection in wild boar, the eradication of the virus was attempted through the culling of the wild host. The goal was to reach a wild boar density at which the reduced contact between infected and susceptible animals would have prevented virus transmission, thus leading the infection to extinction. Although the depopulation strategy is intuitive and direct, the results of its application early in the infected area are invariably negative. The needed hunting effort is hardly, if ever, reached, whereas disturbance causes animals to flee the area, spreading the infection into neighbouring free areas [6,7].

In the recently infected areas of Czech Republic and Belgium, a different eradication approach was applied, which included an immediate hunting ban for the wild boar infected area. Moreover, the eradication strategy consisted of specific actions [8] implemented accordingly to the different phases of the infection [7], and such phases can be identified only through an efficient and continuous passive surveillance based on carcass detection. In the applied strategy it is of paramount importance to define the infected area, defined as the area where carcasses positive to the ASF virus were found, following a planned active search. As finding carcasses in the wild is a demanding task, a model has been developed to optimize the research effort; it describes the habitat characteristics where it is more likely to find wild boar carcasses [9]. However, the model does not address the expected number of carcasses to be detected nor their distribution in time. This information is a function of the R_0_ parameter. R_0_ is the average number of secondary cases caused by one infectious individual during its entire infectious period in a fully susceptible population [10]. It consists of three components: the rate of contact between the number of susceptible and infectious individuals, the probability of transmission, and the duration of the infectiousness. As R_0_ is dependent on several variables both disease- and host population-specific, there is not a unique R_0_ value for an infection, but it is specific for each population. R_0_ is often used to quantify the spread of a disease and as an indicator of the potential magnitude of an epidemic. To calculate R_0_ the number of infected animals for each time step (i.e., day) is needed, but these data are virtually impossible to obtain for a wild population. However, for diseases with a high case–lethality ratio, mortality cases can be used as a proxy for the number of newly infected individuals (e.g., [11]). Once appropriate data on carcasses are collected, several mathematical methods can be used to estimate R_0_ value [12,13,14]. When the value of R_0_ is obtained it can be used to estimate the expected number of carcasses to find within each cycle of infection; the latter is summarised by the doubling time (time needed by the disease to duplicate the number of infected individuals).

Coupling R_0_ and doubling time will allow us to precisely plan the active search of carcasses defining numerical and temporal goals, i.e., how many carcasses should be detected in a certain time window.

The aim of the present paper is to estimate both doubling time and R_0_ from the data obtained during the epidemics observed in Czech Republic and in Belgium. Both the areas are characterised by the typical central European wild boar habitat (managed broadleaf forests producing mast at irregular time intervals) and wild boar hunting practices (sustaining feeding, limited harvesting of adult females, driven hunts during winter months), hence they represent a reference case for countries sharing similar management of both forests and wild boar populations [15,16].

## 2. Materials and Methods

### 2.1. Data Sets

**Figure 1:**
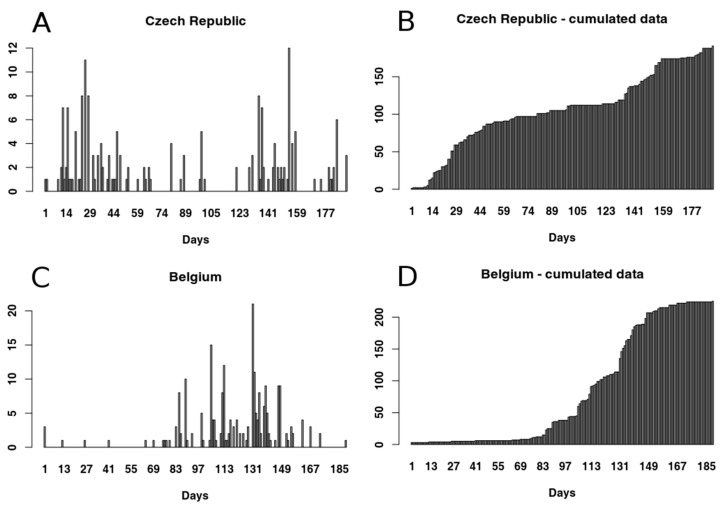
Data sets: (**A**) number of carcasses found in Czech Republic, Zlin area; (**B**) cumulated number of carcasses found in Czech Republic, Zlin area; (**C**) number of carcasses found in Belgium, Virton Forest; (**D**) cumulated number of carcasses found in Belgium, Virton Forest.

Data about PCR (Polymerase Chain Reaction) positive wild boar carcasses have been collected for two infected areas, one in Czech Republic (Zlin area, n = 191), and one in Belgium (Virton Forest, n = 280). Belgium data set contains both fresh and non-fresh carcasses, but for the purpose of this study only fresh carcasses have been considered (n = 225, for the definition of fresh see [7]). Fresh carcasses better represent the pattern of the disease through time, avoiding the bias in the shape of our cumulated data that would have been introduced by including old carcasses. Czech Republic wild boar cases have been recorded during summer and autumn (from 21 June 2017 to 27 December 2017; with a monthly mean temperature ranging from 1.7 °C to 21.8 °C). Belgium cases have been recorded during autumn, winter, and early spring (from 27 September 2018 to 12 April 2019; monthly mean temperature ranging from 0.5 °C to 14.0 °C). Each record of the data sets represents the discovery of a wild boar carcass which resulted positive to real-time PCR (Rt PCR); antibodies detection was not carried out in any of the two areas. Following the finding of the first ASFV positive carcass, usually by chance, carcass search was actively programmed and implemented in both infected areas. For our analysis we grouped data by day of discovery, obtaining a mean of 1.0 ± 2.0 carcasses a day for Czech Republic (range 0–12; Figure 1) and a mean of 1.2 ± 2.8 carcasses a day for Belgium (range 0–21; Figure 1).

### 2.2. R_0_ Estimation Method

Computation of R_0_ was derived from the growth rate of cases [17], an approach already applied to ASF for both wild boar (e.g., in Russia [18]) and domestic pigs (e.g., in Uganda [19] and in Ukraine [20]). The assumption of this approach is that at the beginning of the epidemic, the cumulative distribution of the cases grows at an exponential rate, during which each case produces R_0_ new cases during the infectious period. During the exponential phase of the epidemic, R_0_ can be computed as a function of the doubling time and the duration of the infectious period. This approach requires the following steps: identify the subset of data that follows an exponential growth, estimate the doubling time of the infection event, and then estimate R_0_. For R_0_ estimation, the infectious period was assumed to be 6 days [18,19,20,21,22].

### 2.3. Data Processing

**Figure 2:**
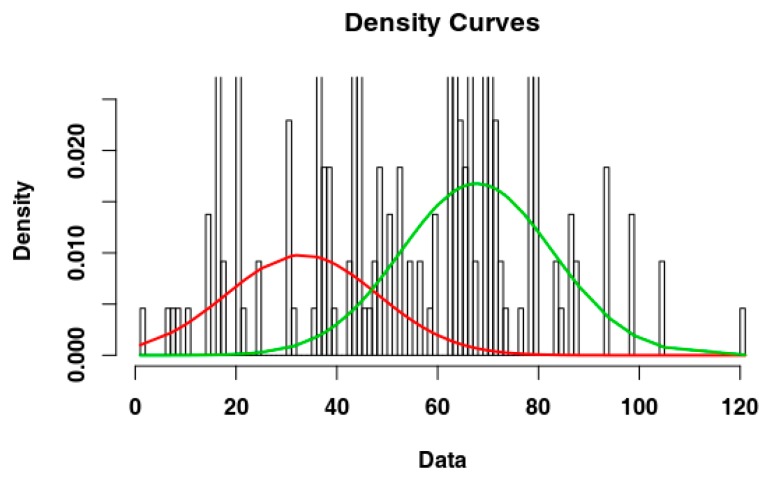
Gaussian Mixed Models on Belgium data set, where we already removed the first 60 days.

Visual exploration of Belgium data suggested that the first part of the data set does not represent the beginning of the epidemic event, i.e., the shape of the cumulative data distribution does not appear to be exponential (see Figure 1D). However, the data set seems to show the presence of a second epidemic event, starting around day 130, therefore we decided to focus our analysis on this data subset. We used Gaussian mixed models (GMM) to test data distribution and verify the presence of two events underlying data distribution (Figure 2). We visually selected the most promising subset for our analysis to be from day 130 onwards.

### 2.4. Identify the Most Suitable Subset of Data

To identify the subset of our data that best fits the exponential distribution we log-transformed our data and fit a linear model. This has been done based on the fact that a logarithmic transformation of an exponential distribution returns a straight line. Therefore, we iteratively fit the linear model to different data subsets, to identify the subset that represents the best fit and use it for our analysis. At each iteration, our procedure subtracted a single record from the end of our data set. Adjusted R-squared and residual sum of squares (RSS) values have been used to evaluate the model fit for each subset.

### 2.5. Doubling Time Calculation

Once the most suitable data subsets have been identified, we used the equation describing the exponential phase of each epidemic to estimate the doubling time for Czech Republic and Belgium. First we used the model formula to calculate the expected number of carcasses (*y*) at day 1 (i.e., *x* = 1), then we doubled that value and solved the model formula for the result (i.e., 2*y*), obtaining the day (*x_t_*) when we expected to find twice the number of carcasses expected at day 1. Then, we calculated the difference between the two *x* values to obtain the doubling time. Since we are using an exponential model, the doubling time value remains constant for any value of *x*.

### 2.6. R_0_ Calculation

R_0_ values have been calculated using the formula from Anderson and May [17]:

Equation (1)

(1) $$1+\frac{ln2}{Td}*D$$

where T_d_ is the doubling time of the epidemic and D is the duration of the infectious period (i.e., 6 days).

## 3. Results

### 3.1. Identify the Most Suitable Subset of Data

#### 3.1.1. Czech Republic

**Figure 3:**
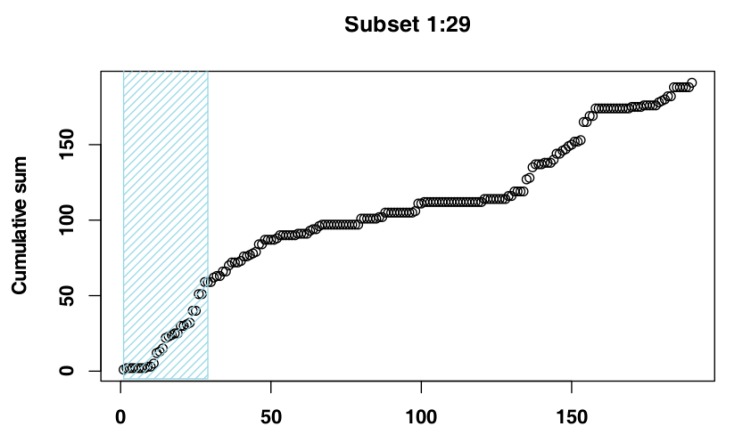
Selected subset for Czech Republic data.

**Figure 4:**
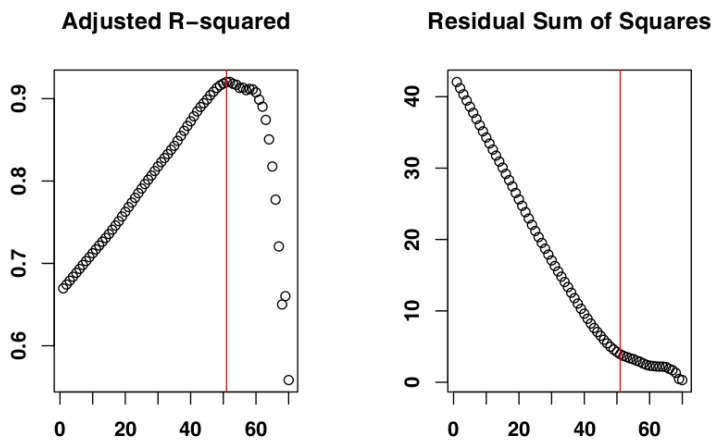
Adjusted R-square and residual sum of squares (RSS) values returned by the iterative procedure applied to Czech Republic data. The red line indicates the values for the selected subset. Numbers on the *x*-axis are the iteration IDs.

**Figure 5:**
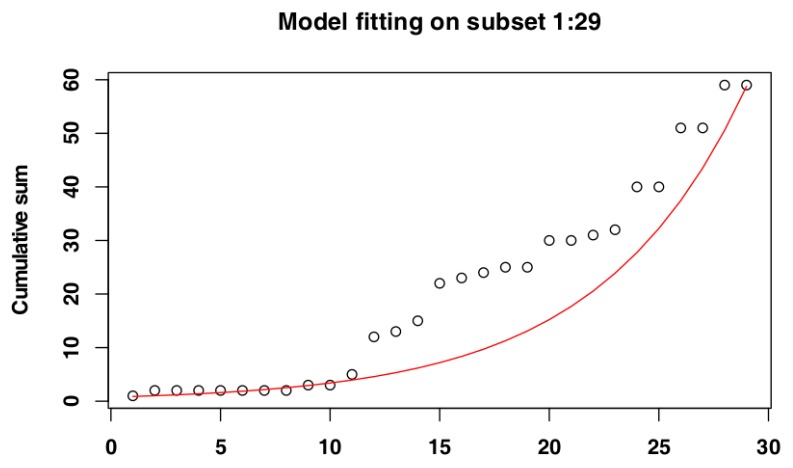
Model fitting for the selected subset for Czech Republic data.

The iterative procedure of model fitting for Czech Republic data identified the subset of days 1–29 (Figure 3) as the one whose cumulative distribution is closest to an exponential distribution, as it returned the highest adjusted R-squared value (Adj-R^2^ = 0.98). The RSS value for this subset (RSS = 4.00) supported its selection as the most suitable subset. The adjusted R-squared values and RSS values returned by the models can be seen in Figure 4 and the model fit to the original data can be seen in Figure 5.

#### 3.1.2. Belgium

**Figure 6:**
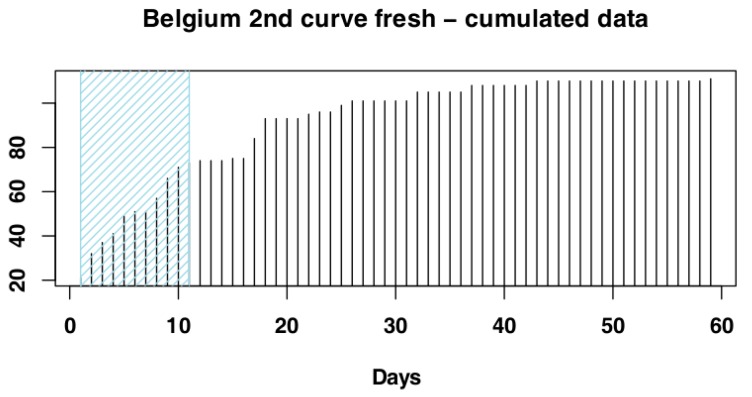
Selected subset for Belgium data.

**Figure 7:**
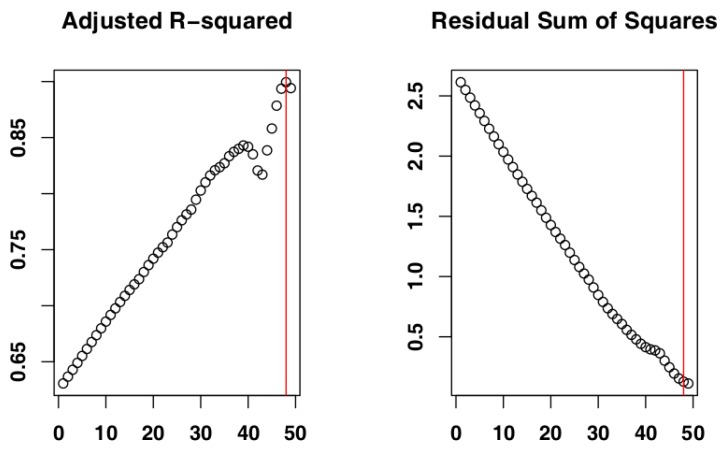
Adjusted R-squared and residual sum of squares (RSS) values for Belgium data. The red line indicates the values returned by the selected subset. Numbers on the *x*-axis are the iteration IDs.

**Figure 8:**
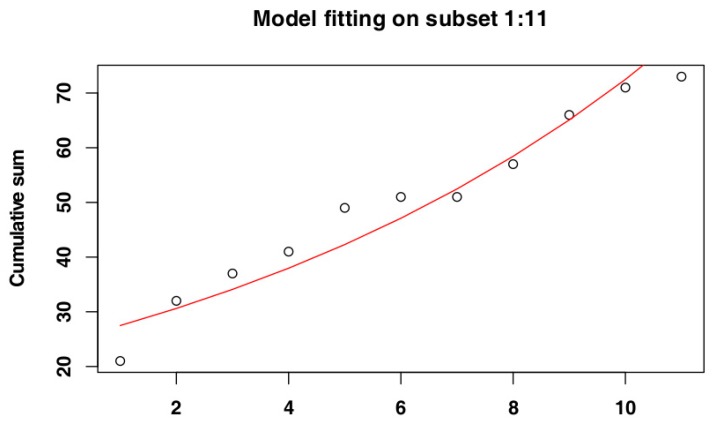
Model fitting for the selected subset for Belgium data.

The iterative procedure for Belgium selected the subset of days 1–11 (Figure 6), as it returned the highest adjusted R-squared value (Adj-R^2^ = 0.9). Again, the RSS value for this subset (RSS = 0.13) supported its selection as the most suitable subset. The adjusted R-squared values and RSS values returned by the models can be seen in Figure 7 and the model fit to the original data can be seen in Figure 8.

### 3.2. Doubling Time and R_0_ Estimation

The doubling time for the selected subset resulted to be 4.39 days for Czech Republic and 6.43 days for Belgium (Table 1). When we resolve Equation (1) for *D* = 6, the resulting R_0_ is 1.95 for Czech Republic and 1.65 days for Belgium (Table 1).

## 4. Discussion

In the two analysed epidemics the infection did not spread to the domestic pig population and thus ASF data refer to the infection in wild boar only. Nonetheless, we consider our results to slightly underestimate actual R_0_ values, since it is unlikely that all carcasses were detected in the field and a few infected animals could have recovered from the disease.

R_0_ values reported in the literature for ASF vary widely, ranging from 0.5 to 18, depending on the type of study conducted: direct or indirect transmission, inter-species or intra-species transmission, within herds or between herds transmission, and field or experimental studies [23]. Our results are comparable to those obtained for between herd transmission (mean value 1.7) or for indirect transmission (mean value 1.5). We consider our estimated R_0_ to result mainly from direct transmission between individuals: at the onset of the infection the wild boar density was high and carcasses were immediately removed and disposed. Wild boar are known to visit carcasses to feed on invertebrates, but only after carcasses have spent some time in the field (e.g., Bassi et al. [24] reported two weeks), so early removal of fresh carcasses strongly reduced the probability of ASF indirect transmission through contact with carcasses, assuming high detectability of carcasses.

Although our R_0_ estimates for the two countries are comparable, the returned value for Belgium is lower than the value for Czech Republic; the difference could be due to the lower wild boar density in Belgium, with respect to Czech Republic. In the two infected areas the disease showed the usually high case–lethality ratio and the culling goal was to depopulate the area, hence the total number of dead animals reported is a good proxy for the real density in the area. After the epidemic, the densities resulted to be 8.5 boars/km^2^ in Czech Republic (Jarosil T., personal communication) and 5.6 boars/km^2^ in Belgium (unpublished Forest Service data). As R_0_ intrinsically contains a contact rate parameter, it is obvious that a change in host density will influence R_0_ value.

As a side note, at the beginning of the epidemic, the hunters of the infected area in Czech Republic reported a wild boar density of about of 3.5 boar/km^2^ (Jarosil T., personal communication).

This underlines the usefulness of R_0_ parameter, as it can describe the expected temporal distribution of cases in a population, while being calculated without the need of information about population size or density [25].

If data collection on carcasses is properly done and starts as soon as the first case is reported, the presented methodology can be easily applied to calculate the specific R_0_, to properly address carcass search, and to better evaluate the infection phase. As an example, the pattern of carcass findings during the first days of the Belgium data set (i.e., first 60 ca. days of the original unfiltered data set) can lead to two interpretations: as the pattern of cumulative cases (i.e., carcasses) does not fit an exponential distribution, it means that either the research area is not properly defined (e.g., too small) or that the infection already passed the very initial epidemic phase. Moreover, comparing the expected and observed temporal distribution of carcass findings it is possible to evaluate the efficiency of the passive surveillance.

## Figures and Tables

**Table 1:** Exponential equations, doubling time values, and R_0_ values for Czech Republic and Belgium epidemics.

	Equation	Doubling Time	R_0_
Czech Rep.	y = e^x*0.158^	4.39	1.95
Belgium	y = e^3.206 + x*0.108^	6.43	1.65
